# Potential Application of *Prunus armeniaca* L. and *P. domestica* L. Leaf Essential Oils as Antioxidant and of Cholinesterases Inhibitors

**DOI:** 10.3390/antiox8010002

**Published:** 2018-12-21

**Authors:** Marco Bonesi, Maria Concetta Tenuta, Monica R. Loizzo, Vincenzo Sicari, Rosa Tundis

**Affiliations:** 1 Department of Pharmacy, Health Science and Nutrition, University of Calabria, Via Pietro Bucci, 87036 Arcavacata di Rende (CS), Italy; marco.bonesi@unical.it (M.B.); mary.tn2006@hotmail.it (M.C.T.); rosa.tundis@unical.it (R.T.); 2 Department of Agricultural Science, Mediterranean University of Reggio Calabria, Via Graziella, Feo di Vito, 89123 Reggio Calabria, Italy; vincenzo.sicari@unirc.it

**Keywords:** *Prunus* species, leaves, essential oil, antioxidant, neuroprotection

## Abstract

The aim of this work is to investigate the in vitro acetylcholinesterase (AChE) and butyrycholinesterase (BChE) inhibitory activities of essential oils obtained by hydrodistillation from the leaves of *Prunus armeniaca* and *P. domestica* in relation to their composition, analysed by Gas Chromatography–Flame Ionization Detector (GC-FID) and Gas Chromatography-Mass Spectrometry (GC-MS) analyses, at different times. Moreover, considering the role of free radicals in the progression of neurodegenerative disorders, the antioxidant properties of essential oils were investigated by using, 2’-azino-bis(3-ethylbenzothiazoline-6-sulphonic acid) (ABTS), 2,2-diphenyl-1-picrylhydrazyl (DPPH), and β-carotene bleaching tests. The relative antioxidant capacity index (RACI) was used to achieve more comprehensive comparison between analysed antioxidant effects of essential oils. *P. armeniaca* oils were more active than *P. domestica* oils against AChE. Against BChE, the most active was the essential oil from *P. domestica* leaves collected in August with an IC_50_ value of 95.80 μg/mL. This oil exerted the highest inhibitory activity of lipid peroxidation with IC_50_ values of 11.15 and 11.39 μg/mL after 30 and 60 min of incubation, respectively. All samples demonstrated a remarkable ABTS radicals scavenging activity, with IC_50_ values in the range 0.45–0.57 μg/mL in comparison to the positive control, ascorbic acid.

## 1. Introduction

The prevalence of neurodegenerative disorders is increasing. Alzheimer’s disease (AD) is one of the most common neurodegenerative disease characterized by cognitive impairment and gradual memory loss [1]. Anomalous deposition of amyloid plaques and neurofibrillary tangles (NFTs) in different brain regions, deficit of cholinergic neurotransmission, inflammation, and oxidative stress, are some of the pathways involved in the development and progression of AD [2]. Amyloid plaques are constituted by Aβ peptides originating from amyloid precursor protein (APP) by β- and γ-secretase enzymes [3]. Aβ peptides polymerize, forming the insoluble filaments that constitute senile plaques.

Neuro-inflammation processes, reduced cholinergic neurotransmission, microglial activation, and cytokine release also occur [4]. As stated by the cholinergic hypothesis, the reduced cholinergic neurotransmission in the cerebral cortex and the destruction of cholinergic neurons disturb cognitive function [5]. Depletion of the levels of acetylcholine has been observed in patients affected by dementia and in vivo studies have confirmed the role of acetylcholine in learning and memory. Thus, acetylcholinesterase (AChE) enzyme is an important target to improve the cholinergic deficit that characterizes AD [6]. Another cholinesterase, such as butyrylcholinesterase (BChE, has been identified in humans and showed an auxiliary role in synaptic transmission. Its expression is higher in the late stage of AD.

Different in vitro and in vivo works demonstrated the occurrence of oxidative events in the course of AD that support the important role of oxidative stress in this pathology. In fact, it was reported that oxidative stress is linked with mitochondria dysfunction, Aβ-induced neuronal loss, tau protein pathology, and metal homeostasis disturbance [7]. Oxidative stress produces depletion of brain antioxidants levels, including vitamin C, uric acid, vitamin E, and antioxidant enzymes, including superoxide dismutase, catalase, and glutathione reductase [8]. Lipid peroxidation is enhanced in AD. The most common lipid peroxidation products found in AD patients are isoprostanoids and reactive aldehydes, such as malondialdehyde (MDA), 4-hydroxynonal, and 2-propenal. Several plant extracts and pure naturally occurring compounds, including alkaloids, polyphenols, and terpenes, have been investigated as potential new neuroprotective agents. Natural cholinesterase inhibitors, such as rivastigmine and galantamine, and *N*-methyl-d-aspartate receptor antagonists, such as memantine, are actually used for the treatment of AD. Besides anti-cholinesterases properties, most natural inhibitors of AChE and BChE have generally showed additional biological activities, principally antioxidant activity. This enables these phytochemicals to be applied as a multi-target strategy against AD. In this regard, essential oils, constituted mainly by monoterpenes hydrocarbon, sesquiterpenes hydrocarbon, oxygenated sesquiterpenes, oxygenated monoterpenes, and esters, has recently demonstrated antioxidant and neuroprotective effects [9,10].

Following our previous studies in which we have investigated the potential use of different essential oils from *Citrus*, *Salvia*, *Cistus*, and *Pinus* species for the treatment of AD [11,12,13,14], herein, we analysed essential oils from the leaves of *Prunus armeniaca* and *P. domestica* collected at three different times (June, July, and August). In particular, the objectives of this study were: (i) To investigate the ability of *P. armeniaca* and *P. domestica* essential oils to inhibit AChE and BChE enzymes; (ii) to determine the antioxidant properties by using three different in vitro tests, such as 2,2’-azino-bis(3-ethylbenzothiazoline-6-sulphonic acid) (ABTS), 2,2-diphenyl-1-picrylhydrazyl (DPPH), and β-carotene bleaching assays; and (iii) to evaluate the correlation of the chemical profile with the activities.

## 2. Materials and Methods

### 2.1. Chemicals and Reagents

Sodium phosphate buffer, acetylcholinesterase from *Electrophorus electricus* (EC 3.1.1.7, Type VI-S), butyrylcholinesterase from equine serum (EC 3.1.1.8), Tween 20, anhydrous sodium sulphate, ascorbic acid, 2,2-diphenyl-1-picrylhydrazyl (DPPH), 5,5’-dithiobis(2-nitrobenzoic-acid) (DTNB), acetylthiocholine iodide (ATCI), butyrylthiocholine iodide (BTCI), propyl gallate, β-carotene, physostigmine, and 2,2’-azino-bis(3-ethylbenzothiazoline-6-sulphonic acid) (ABTS) were acquired from Sigma-Aldrich S.p.a. (Milan, Italy). All other reagents were obtained from VWR International s.r.l. (Milan, Italy). All solvents used in this study were of analytical grade.

### 2.2. Plant Materials and Essential Oils’ Preparation

*Prunus armeniaca* (P1–P3) and *P. domestica* (P4–P6) leaves were collected in Cosenza (Italy) in three months, such as June (P1, 1396 g; P4, 1892 g), July (P2, 937 g; P5, 920 g), and August (P3, 900 g; P6, 810 g) 2013, and authenticated at the Natural History Museum of Calabria and Botanic Garden, University of Calabria (Rende, Cosenza, Italy). Leaves were subjected to hydrodistillation for 3 h, by using a Clevengertype apparatus as previously reported [14]. *P. armeniaca* (1.5, 1.2, and 1.0 mL for P1, P2, and P3, respectively) and *P. domestica* (1.3, 1.5, and 2.0 mL for P4, P5, and P6, respectively) essential oils were stored at +4 °C in a brown bottle under N_2_ until analysed for their chemical profile and tested for their bioactivity.

### 2.3. Gas Chromatography (GC-FID) and Gas Chromatography-Mass Spectrometry (GC-MS) Analyses

*P. armeniaca* and *P. domestica* essential oils were analysed by GC-FID and GC-MS. GC-MS analyses were performed by using a Hewlett-Packard 6890 gas chromatograph (Agilent, Milan, Italy) with a HP-5 MS capillary column (30 m length, 0.25 mm i.d., 0.25 μm film thickness) (Agilent, Milan, Italy) interfaced with a Hewlett Packard 5973 Mass Selective (EI, 70 eV) (Agilent, Milan, Italy), by using helium as the carrier gas with a flow of 1.0 mL/min. The following analytical conditions were used: 3 min at 50 °C, then 50–280 °C at a rate of 13 °C/min; then 10 min at 280 °C. GC analysis was carried out on a Shimadzu GC17A (Shimadzu, Milan, Italy) equipped with a FID (Flame Ionization Detector) (Shimadzu, Milan, Italy). Essential oils were analysed on a HP-5 MS capillary column (30 m length, 0.25 mm i.d., 0.25 μm film thickness) (Agilent, Milan, Italy), by using nitrogen as the carrier gas at a constant flow of 1.0 mL/min and a split ratio of 1:30. Analytical conditions were: 3 min at 50 °C, then 50–280 °C at a rate of 13 °C/min; then 10 min at 280 °C. Injector and detector temperatures were maintained at 250 and 280 °C, respectively. Compounds’ identification was made by comparing their mass spectral data with the Wiley 275 library and by referring to compounds available in our laboratory and/or known in the literature. Quantitative determinations were carried out by peak area normalization by using an external standard method. Data are reported in Table 1.

### 2.4. In Vitro Antioxidant Activity (DPPH, ABTS, β-Carotene Bleaching Tests)

To determine the free radicals scavenging activity of *P. armeniaca* and *P. domestica* essential oils, 2,2’-diphenypicryl hydrazyl (DPPH) and 2,2’-azino-bis(3-ethylbenzothiazoline-6-sulphonic acid) (ABTS) tests were employed testing different essential oils concentrations in the range of 62.5–100 μg/mL and 25–400 μg/mL for DPPH and ABTS test, respectively [15]. Ascorbic acid was employed as positive control.

The ability of essential oils to inhibit lipid peroxidation was explored by using the β-carotene bleaching test as previously reported [15]. Essential oils concentrations in the range of 6.25–100 μg/mL were tested. Measurements were carried out at initial time (*t* = 0 min), and after 30 and 60 min of incubation. Propyl gallate was used as a positive control.

### 2.5. RACI Determination

Relative antioxidant capacity index (RACI) was calculated. RACI is a statistical application that integrates antioxidant results obtained by using different in vitro tests and provides a reasonably accurate ranking of the antioxidant ability of investigated samples. Herein, standard scores were obtained from data from the DPPH, ABTS, and β-carotene bleaching tests without unrestricted units and no variance between the applied methods. The standard score was calculated by using the following equation: (x − µ)/σ, where x is the raw data, µ is the mean, and σ is the standard deviation [16].

### 2.6. Bioassay for Acetylcholinesterase (AChE) and Butyrylcholinesterase (BChE) Inhibitory Activity

Acetylcholinesterase (AChE) and butyrylcholinesterase (BChE) inhibitory activity was evaluated by Ellman’s method as previously described [13]. The enzyme (AChE or BChE), *P. armeniaca* and *P. domestica* essential oils, and buffer were pre-incubated for 30 min at 4 °C. The addition of ATCI or BTCI and DTNB solution started the reaction. Then, the reaction was halted by adding physostigmine in plates placed in an ice bath. ATCI or BTCI hydrolysis was spectrophotometrically recorded at 405 nm and the percentage inhibition was calculated.

### 2.7. Statistical Analysis

IC_50_ values (concentrations giving 50% inhibition) were calculated by nonlinear regression by using Prism GraphPad Prism version 4.0 for Windows (GraphPad Software, San Diego, CA, USA).

One-way analysis of variance test (ANOVA) followed by a multicomparison Dunnett’s test were used.

## 3. Results and Discussion

### 3.1. Chemical Composition of Essential Oils

To identify active compounds of *P. armeniaca* and *P. domestica* essential oils, gas chromatography systems were employed. In total, 23 main volatiles were identified.

As shown in Table 1, *P. armeniaca* fruits were characterized mainly by phytol (19.42–19.92%), manoyl oxide (5.21–6.53%), linalool (4.44–4.81%), limonene (2.44–2.87%), and (*E*)-2-hexenal (3.54–4.87%). The two alkanes, nonacosane (21.11–23.76%) and heptacosane (10.14–11.61%) were also abundant. The essential oil from *P. armeniaca* leaves collected in June (P1) showed high percentages of γ-cadinene (4.76%), δ-cadinene (4.73%), and pentacosane (7.39%) in comparison to the other *P. armeniaca* oils P2 and P3.

*P. domestica* leaves showed pentacosane (15.78–16.83%), phytol (25.83–9.36%), hexadecanoic acid (6.33–7.67%), and benzaldehyde (5.66%) as the most abundant constituents. Other alkanes identified in high percentages were tricosane, heptacosane, and nonacosane. Some differences can be highlighted in the three months of collection (samples P4–P6). Indeed, the essential oil, P4, showed the presence of of γ-cadinene (0.71%) and δ-cadinene (0.42%), identified in trace in the other two samples, P5 and P6. Moreover, the essential oil, P6, was characterized by the presence of *p*-mentha-2,4(8)-diene and *trans*-β-farnesene, and entriacontane, identified in trace in the other two oils.

The comparative analysis of essential oils from both *Prunus* species revealed the presence of (*E*)-2-hexenal, limonene, linalool, (*E*)-β-ionone, and manoyl oxide only in *P. armeniaca* oils. On the other hand, benzaldehyde, *p*-mentha-2,4(8)-diene, *trans*-β-farnesene, tetradecanoic acid, hexadecanoic acid, triacontane, and entriacontane were identified only in *P. domestica* essential oils.

According to our knowledge, the literature reveals very few studies on essential oils from the leaves of *P. armeniaca* and *P. domestica*.

Only one study has reported the composition of the essential oil from *P. domestica* leaves [17]. Based on the results obtained in this work, *P. domestica* leaves are characterized by benzaldehyde as the dominant constituent. Instead, the volatile components of *P. armeniaca* fruits have been extensively investigated [18,19].

### 3.2. Bioactivities

DPPH, ABTS, and β-carotene bleaching tests were used to investigate the antioxidant potential of *P. armeniaca* and *P. domestica* essential oils. A concentration-effects relationship was found for all essential oils (Table 2). ABTS and DPPH assays were employed to analyse the radicals scavenging activity.

A very interesting ABTS radicals scavenging activity was found for all analysed oils with Inhibitory Concentration 50% (IC_50_) values in the range 0.45–0.54 μg/mL in comparison to the positive control, ascorbic acid (IC_50_ value of 1.70 μg/mL).

In the DPPH test, the most active sample was the essential oil from *P. domestica* leaves collected in July (P5) with an IC_50_ value of 73.78 μg/mL. The other IC_50_ values were in the range of 80.05–105.76 μg/mL. The different antioxidant activity demonstrated in these tests by the essential oils may be related to the different stereochemistry and training mechanism of the ABTS and DPPH radical. For this reason, after reaction with antioxidant compounds, these radicals gave a different response [20].

*P. domestica* essential oils were more active than *P. armeniaca* essential oils in inhibiting lipid peroxidation using the β-carotene-linoleic acid test system. IC_50_ values in the range of 11.15–14.75 μg/mL and 11.39–16.19 μg/mL after 30 and 60 min of incubation were found for the essential oils, P4–P6.

**Figure 1:**
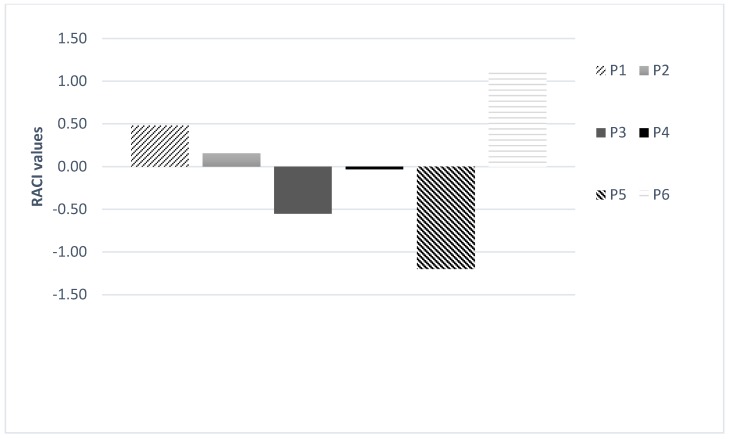
Relative Antioxidant Capacity Index (RACI) value of *P. armeniaca* (P1–P3) and *P. domestica* (P4–P6) essential oils.

The statistical approach, Relative Antioxidant Capacity Index (RACI), was used to identify the sample characterized by the highest antioxidant potential. Based on this index, sample P5 showed the highest antioxidant potency (Figure 1).

AChE and BChE inhibitory activity were used to investigate the potential neuroprotective activity of *P. domestica* and *P. armeniaca* essential oils. A concentration-response relationship was observed for all tested essential oils. Table 3 reports the IC_50_ values.

Against AChE, the best activity was exerted by *P. armeniaca* essential oils, particularly by P1 with an IC_50_ value of 97.60 μg/mL. This oil was characterized by the presence of phytol, δ-cadinene, γ-cadinene, linalool, limonene, and (*E*)-2-hexenal as dominant constituents.

The most active in inhibiting BChE were *P. domestica* P6 and P5 oils with IC_50_ values of 95.80 and 100.20 μg/mL, respectively. The other IC_50_ values were in the range 138.30–226.90 μg/mL.

One of the dominant compounds that characterized the essential oil of *P. armeniaca* and *P. domestica* was phytol (3,7,11,15-tetramethylhexadec-2-en-1-ol). Phytol is a diterpene product of chlorophyll metabolism. Therefore, it is a compound abundant in nature. In a previous work [21], phytol has been demonstrated to reduce the production of free radicals. Phytol revealed a strong antioxidant activity due to its ability to remove hydroxyl radicals and could prevent lipid peroxidation by inhibiting the amount of Thiobarbituric Acid Reactive Species (TBARS), inhibiting cell damage caused by free radicals. Similar results were obtained with Trolox, used as a positive control. Syad et al. [22] showed an IC_50_ value for phytol of 95.27 μg/mL in the DPPH test. The same research group analysed the potential cholinesterases’ inhibitory activity of this diterpene.

Phytol inhibited AChE and BChE with IC_50_ values of 2.70 and 5.79 μg/mL against AChE and BChE, respectively. Different studies have demonstrated the neuroprotective activity of terpenes. Monoterpenes were mainly investigated. IC_50_ values of 0.63 and 0.87 mM were found for α-pinene against AChE [23] and BChE [12], respectively. Previously, Miyazawa et al. [24] reported the ability of linalool to inhibit AChE with a percentage of 37% at 164 μg/mL. Successively, Menichini et al. [25] showed IC_50_ values of 225.9 and 456.2 μg/mL for limonene against AChE and BChE, respectively.

## 4. Conclusions

In summary, this study reports for the first time the antioxidant and neuroprotective effects of the essential oils obtained by hydrodistillation from the leaves of *P. armeniaca* and *P. domestica*. Our results demonstrated a very interesting radical scavenging activity in the ABTS test by all investigated essential oils. *P. armeniaca* essential oils, particularly P1, were active in inhibiting AChE. On the other hand, *P. domestica* P6 and P5 were the most active in inhibiting BChE.

One of the dominant compounds that characterized essential oils of both *P. armeniaca* and *P. domestica* leaves was phytol, a diterpene commonly found in plants, which has previously been demonstrated to be an interesting antioxidant and neuroprotective compound. However, the unique composition of the essential oils allows for a synergistic action between individual compounds and is responsible for the found bioactivity. The obtained results underline the potential health benefits of the studied essential oils and suggest their potential use for the formulation of new products for the treatment of neurodegenerative diseases.

## Figures and Tables

**Table 1:** The main volatiles of *P. armeniaca* (P1–P3) and *P. domestica* (P4–P6) leaves essential oils.

Compound	RI ^a^	Relative Amount (%)					
		P1	P2	P3	P4	P5	P6
(*E*)-2-Hexenal	854	3.54 ± 0.21	4.44 ± 0.42	4.87 ± 0.40	-	-	-
α-Pinene	938	0.64 ± 0.01	0.58 ± 0.02	0.42 ± 0.06	0.23 ± 0.02	0.18 ± 0.01	0.16 ± 0.01
Benzaldehyde	963	-	-	-	5.66 ± 0.3	5.06 ± 0.20	4.32 ± 0.12
β-Pinene	980	0.76 ± 0.02	0.77 ± 0.02	0.65 ± 0.01	tr	tr	tr
Limonene	1030	2.54 ± 0.03	2.44 ± 0.02	2.87 ± 0.05	-	-	-
Linalool	1098	4.54 ± 0.01	4.44 ± 0.05	4.81 ± 0.02	-	-	-
*p*-Mentha-2,4(8)-diene	1142	-	-	-	tr	tr	2.01 ± 0.32
*trans*-Caryophyllene	1415	0.20 ± 0.01	0.18 ± 0.02	0.15 ± 0.01	0.13 ± 0.01	0.15 ± 0.02	0.16 ± 0.03
*trans*-β-Farnesene	1441	-	-	-	tr	tr	2.13 ± 0.22
(*E*)-β-Ionone	1484	1.22 ± 0.11	1.12 ± 0.1	1.14 ± 0.50	-	-	-
γ-Cadinene	1515	4.76 ± 0.80	0.67 ± 0.03	0.70 ± 0.02	0.71 ± 0.04	tr	tr
δ-Cadinene	1526	4.73 ± 0.75	0.78 ± 0.07	0.56 ± 0.03	0.42 ± 0.02	tr	tr
Tetradecanoic acid	1768	-	-	-	1.52 ± 0.5	1.45 ± 0.14	1.40 ± 0.22
(*Z*)-Phytol	1950	19.92 ± 1.52	19.88 ± 2.1	19.42 ± 1.84	25.83 ± 2.6	12.81 ± 1.4	9.36 ± 1.10
Hexadecanoic acid	1969	-	-	-	7.67 ± 0.84	7.58 ± 1.23	6.33 ± 1.20
Manoyl oxide	1989	6.53 ± 1.10	5.45 ± 1.13	5.21 ± 1.23	-	-	-
Tricosane	2300	2.30 ± 0.34	2.30 ± 0.55	2.39 ± 0.42	6.14 ± 0.02	6.33 ± 0.20	8.55 ± 0.92
Tetracosane	2400	1.97 ± 0.21	2.02 ± 0.11	2.06 ± 0.20	9.13 ± 1.6	8.88 ± 0.02	4.82 ± 0.94
Pentacosane	2500	7.39 ± 0.64	2.28 ± 0.20	3.93 ± 0.31	16.83 ± 1.3	16.30 ± 0.02	15.78 ± 1.7
Heptacosane	2700	10.14 ± 1.21	10.45 ± 1.7	11.61 ± 1.45	8.94 ± 0.20	4.58 ± 0.40	3.99 ± 0.23
Nonacosane	2900	21.76 ± 1.53	21.25 ± 1.1	21.11 ± 1.42	11.08 ± 1.60	3.86 ± 0.63	3.68 ± 0.42
Triacontane	3000	-	-	-	2.21 ± 0.26	2.11 ± 0.52	5.06 ± 0.71
Entriacontane	3100	-	-	-	-	-	4.06 ± 0.31

P1 and P4: essential oils from leaves collected in June; P2 and P5: essential oils from leaves collected in July; P3 and P6: essential oils from leaves collected in August. RI: Retention index. Data are reported as mean ± standard deviation (*n* = 3). ^a^ Retention indices on HP 5MS column; Compounds identification: comparison of retention times, comparison of mass spectra with mass spectrometry libraries, comparison with authentic compounds; tr: trace (<0.1%). -: not detected.

**Table 2:** Antioxidant activity (IC_50_, µg/mL) evaluated by 2,2-diphenyl-1-picrylhydrazyl (DPPH), 2,2’-azino-bis(3-ethylbenzothiazoline-6-sulphonic acid) (ABTS), β carotene-bleaching test and Relative Antioxidant Capacity Index (RACI) values.

Essential oil	DPPH Test	ABTS Test	β-Carotene-Bleaching Test		RACI
*P. armeniaca*					
P1	83.86 ± 1.25 *	0.47 ± 0.07 *	28.33 ± 0.48 *	20.73 ± 0.54 *	0.48
P2	105.76 ± 2.81 *	0.54 ± 0.06 *	23.05 ± 0.65 *	18.55 ± 0.57 *	0.16
P3	80.05 ± 2.44 *	0.57 ± 0.04 *	22.64 ± 0.46 *	17.30 ± 0.47 *	−0.55
*P. domestica*					
P4	88.33 ± 1.90 *	0.45 ± 0.07 *	14.75 ± 0.56 *	16.19 ± 0.54 *	−0.03
P5	73.78 ± 1.64 *	0.48 ± 0.05 *	14.68 ± 0.51 *	13.07 ± 0.70 *	−1.20
P6	103.32 ± 3.26 *	0.50 ± 0.04 *	11.15 ± 0.43 *	11.39 ± 0.53 *	1.10
Positive control					
Ascorbic acid	5.01 ± 0.83	1.70 ± 0.11			
Propyl gallate			1.0 ± 0.04	1.0 ± 0.03	

Data are expressed as mean ± S.D. (*n* = 3); DPPH Radical Scavenging Activity Assay; Antioxidant Capacity Determined by Radical Cation (ABTS^+^); β-Carotene bleaching test. Differences within and between groups were evaluated by one-way analysis of variance test followed by a multicomparison Dunnett’s test. DPPH: * *p* < 0.01; ABTS: * *p* < 0.01; β-carotene-bleaching test: * *p* < 0.01.

**Table 3:** Acetylholinesterase (AChE) and butyrylcholinesterase (BChE) inhibitory activity of *P. armeniaca* and *P. domestica* leaves essential oils (IC_50_ μg/mL).

Essential Oils	AChE	BChE
*P. armeniaca*		
P1	97.60 ± 1.94 *	138.30 ± 1.13 *
P2	98.20 ± 1.51 *	210.80 ± 2.50 *
P3	98.50 ± 1.96 *	226.90 ± 3.31 *
*P. domestica*		
P4	98.60 ± 2.20 *	209.70 ± 1.82 *
P5	135.10 ± 3.45 *	100.20 ± 1.42 *
P6	171.80 ± 3.63 *	95.80 ± 1.60 *
Positive control		
Physostigmine	0.17 ± 0.01	2.40 ± 0.02

Data are expressed as mean ± S.D. (*n* = 3); AChE: Acetylcholinesterase assay; BChE: Butirrilcholinesterase assay. Differences within and between groups were evaluated by one-way analysis of variance test followed by a multicomparison Dunnett’s test. AChE: * *p* < 0.01; BChE: * *p* < 0.01.
